# Adjuvant effects of a sequence-engineered mRNA vaccine: translational profiling demonstrates similar human and murine innate response

**DOI:** 10.1186/s12967-016-1111-6

**Published:** 2017-01-03

**Authors:** Darin K. Edwards, Edith Jasny, Heesik Yoon, Nigel Horscroft, Brian Schanen, Tanya Geter, Mariola Fotin-Mleczek, Benjamin Petsch, Vaughan Wittman

**Affiliations:** 1 Sanofi Pasteur, VaxDesign Campus, 2501 Discovery Drive Suite 300, Orlando, FL USA; 2 CureVac AG, Paul-Ehrlich-Str. 15, 72076 Tübingen, Germany

**Keywords:** mRNA, Vaccine, Innate, In vitro, MIMIC, Adjuvant, Human, Mouse, RLRs, CLRs, TLRs, Self-adjuvantation

## Abstract

**Background:**

Prophylactic and therapeutic vaccines often depend upon a strong activation of the innate immune system to drive a potent adaptive immune response, often mediated by a strong adjuvant. For a number of adjuvants immunological readouts may not be consistent across species.

**Methods:**

In this study, we evaluated the innate immunostimulatory potential of mRNA vaccines in both humans and mice, using a novel mRNA-based vaccine encoding influenza A hemagglutinin of the pandemic strain H1N1pdm09 as a model. This evaluation was performed using an in vitro model of human innate immunity and in vivo in mice after intradermal injection.

**Results:**

Results suggest that immunostimulation from the mRNA vaccine in humans is similar to that in mice and acts through cellular RNA sensors, with genes for RLRs [*ddx58* (RIG-1) and *ifih1* (MDA-5)], TLRs (*tlr3*, *tlr7*, and *tlr8*-*human only*), and CLRs (*clec4gp1*, *clec2d*, *cledl1*) all significantly up-regulated by the mRNA vaccine. The up-regulation of TLR8 and TLR7 points to the involvement of both mDCs and pDCs in the response to the mRNA vaccine in humans. In both humans and mice activation of these pathways drove maturation and activation of immune cells as well as production of cytokines and chemokines known to attract and activate key players of the innate and adaptive immune system.

**Conclusion:**

This translational approach not only allowed for identification of the basic mechanisms of self-adjuvantation from the mRNA vaccine but also for comparison of the response across species, a response that appears relatively conserved or at least convergent between the in vitro human and in vivo mouse models.

**Electronic supplementary material:**

The online version of this article (doi:10.1186/s12967-016-1111-6) contains supplementary material, which is available to authorized users.

## Background

Despite evidence demonstrating that predictive immunological parameters may not be applicable across species, preclinical assessments of vaccines or vaccine adjuvants still typically rely on mouse models as the experimental tool of choice [1, 2]. Significant differences have been demonstrated between mouse and human immune system development, activation and response to challenge [2–5]. These differences have led to failure in clinical trials of formulations that appeared promising in preclinical studies [2–6]. Because a mouse cannot be considered a “small human” the development of better methods for the study and analysis of human-based immune system models has been identified as an area of critical need in vaccinology. The need for human-based methods has begun to be filled with the continued development in vitro assays [7, 8] and humanized mice (HM) that harbor a human immune system [9, 10], both of which show promise but neither of which are currently widely used. Human-based in vitro models may not always replicate the entire immunomodulatory activity of an adjuvant or vaccine and are thus a logical complement to in vivo studies [1]. This combination of in vitro and in vivo models offers the opportunity to identify cellular receptors and pathways that are conserved between mice and humans as well as species-specific differences in innate and adaptive immune response to vaccines and vaccine adjuvants.

New in vitro technologies for the pre-clinical assessment of innate response to vaccines or adjuvants have been developed. These include new human-based assays that utilize human monocytoid cell lines or primary immune cells to detect the innate response and safety profile of pyrogens, toxic compounds, adjuvants, and vaccines [11–15]. One such model, the Modular Immune In vitro Construct (MIMIC®), models human innate and adaptive immunity in a sensitive, automated, and cost-effective manner [16]. Two distinct modules of the MIMIC®, the Peripheral Tissue Equivalent model (PTE) and Transwell Peripheral Tissue Equivalent model (TW-PTE), are biomimetic modules designed to simulate innate immune response as it occurs in peripheral tissues such as the skin following an encounter with a vaccine or a pathogen, and can be used to examine human responses against vaccines or vaccine adjuvants. They utilize primary human immune cells coupled with naturally occurring signaling processes to replicate the development of cells responsible for much of the innate immune response. These modules have been shown to reflect appropriate cellular profiles (programmed death, cytokine production, and antigen presenting cell activation/maturation) following stimulation by a variety of test agents including monoclonal antibodies (e.g. TGN1412), seasonal influenza vaccines, immunomodulators and immunosuppressants such as TLR agonists and cyclosporine, respectively [11, 13, 16–19]. Genome-wide transcriptome analysis represents an additional tool for the evaluation of innate response to vaccines or vaccine adjuvants, providing a signature of innate immune response to various challenges [1, 20–22]. Molecular signatures in the blood of humans induced a few days after vaccination have been used to predict the magnitude of later immune responses to a vaccine and are beginning to yield insights about the nature of the innate and adaptive responses to vaccination [23–25]. Additionally, in the vein of translational science, this technology can be applied to the evaluation of vaccine adjuvants in pre-clinical assessments including both in vivo models [e.g. murine, non-human primates (NHP)] and in vitro models (e.g. MIMIC®), the results of which have direct applications to later clinical evaluations in humans.

In recent years researchers have begun developing new classes of vaccine adjuvants which target natural innate response pathways in immune cells. These include compounds targeting pattern recognition receptors (PRRs) such as the TLRs and RLRs. PRR agonists have garnered considerable interest in recent years based on their ability to activate an immune response in a manner consistent with that triggered by invading pathogens. For example, the use of the synthetic ligand CpG (a TLR9 agonist) co-administered with various protein antigens has been investigated in a number of preclinical trials, and shown to induce potent antigen-specific responses [26–29]. Activation of PRRs leads to downstream activation of transcription factors resulting in expression of various genes that drive immune cell maturation, expression of co-stimulatory molecules and production of cytokines and chemokines [30–38]. Viral single-stranded RNA (ssRNA) and double-stranded RNA (dsRNA) are among the many PRR-selective agonists binding and activating TLRs, RLRs, and CLRs in cellular membranes, endosomal compartments, and inside the cell through cytoplasmic sensors [32–34, 38]. Species-specific response to ssRNA from human immunodeficiency virus (HIV) has been demonstrated between mice and human, with murine TLR7 and human TLR8 mediating recognition of GU-rich ssRNA, respectively [31, 33]. In both mouse and human, however, responses to ssRNA and dsRNA through TLRs and RLRs converge on NF-κB and mitogen-activated protein kinase signaling pathways, including the TLR, interleukin (IL)-1, and c-jun N-terminal kinase (JNK) pathways [32]. Activation of these pathways in multiple immune cell subsets including pDCs, mDCs, monocytes, B-cells, and T-cells results in up-regulation of innate stimulation pathways [30–37].

Synthetic nucleic acids vaccines are being investigated as alternatives to traditional vaccines. When used as vaccines nucleic acids have the potential to not only trigger an immunogenic response to the antigens they encode but also to trigger innate sensors of ribonucleic acid (RNA) or deoxyribonucleic acid (DNA) in immune cells. Although DNA-based vaccines have been investigated for use in molecular medicine and vaccinology, clinical applications have increasingly become compromised based on the efficacy and potential risks inherent in use of plasmid DNA. As an alternative to DNA, mRNA based vaccines have been developed to take advantage of the fact that mRNA molecules have the ability to transiently encode immunogenic antigens and also possess self-adjuvanting activity [39]. A new class of mRNA vaccines, RNActive® vaccines, is based on conventional mRNA molecules that have been engineered and sequence-modified to optimize various aspects of the molecule, leading to enhanced mRNA half-life and protein expression [40]. An important building block for the formulation of RNActive® vaccines is protamine, a cationic peptide that forms complexes with RNA. RNActive® vaccines containing protamine consists of an engineered mRNA, which in part is complexed with protamine. This formulation combines the strong expression profile of optimized mRNA with enhanced immune stimulation induced by protamine-complexed mRNA reported to activate the TLR7 receptor [40–43]. This self-adjuvant activity has been investigated in pre-clinical animal studies as well as in clinical safety investigations for both therapeutic cancer and prophylactic vaccines [40, 42–46].

In the present study, we applied a combined series of analytical techniques which constituted an original translational approach to a pre-clinical assessment of the basic mechanisms of self-adjuvantation from mRNA vaccines in an in vitro human model and in vivo in inbred mice. We used the in vitro model of the human immune system termed the MIMIC® to evaluate innate responses induced by distinct doses of the mRNA vaccine encoding influenza A hemagglutinin (HA). These responses were compared to profiles found in C57 BL/6-mice after intradermal injection. The murine samples were taken from two locations, at the injection site and in the draining lymph node (dLN). Phenotypic alterations of immune stimulatory cells and cytokine response in both the MIMIC® and in the mice were analyzed and compared. In each case an analysis of transcriptional changes was also performed, with activation pathways evaluated to compare gene expression profiles in both the human MIMIC® and the mouse after intradermal injection (ID) with mRNA vaccine.

## Methods

### Study design

This study was designed to evaluate the innate stimulatory profiles and basic mechanisms of self-adjuvantation of an mRNA-based vaccine encoding influenza A hemagglutinin in humans and inbred C57BL/6 mice. For all three phases of this study an mRNA vaccine encoding influenza A hemagglutinin of the pandemic strain H1N1pdm09 from the isolate A/Netherlands/602/2009 was used as the model [43, 45]. The first study phase consisted of experiments on MIMIC®-PTE modules to assess the adjuvant properties of different concentrations of mRNA vaccine versus a benchmark vaccine (Fluzone®, Sanofi Pasteur) and the TLR7/8 agonist R848. These experiments were designed to test the immunostimulatory potential of these treatments in humans through the use of the human MIMIC® system to establish associated phenotypic and cytokine profiles. The second study phase was an analysis of transcriptome changes in humans in response to immunostimulation with the mRNA vaccine or R848 as a positive control of TLR7/8 activation. For this study the human MIMIC® Transwell-PTE module was used to generate RNA samples for use in full genome microarray analysis. The third and final phase of this work was performed in wild type mice to assess the mechanisms of self-adjuvantation from the mRNA vaccine by evaluation of cellular and molecular sensors at the injection site and in the dLN. To perform this analysis of gene expression patterns a full genome microarray analysis was performed on skin biopsies or the dLN after intradermal injection of the mRNA vaccine.

### Preparation of peripheral blood mononuclear cells for MIMIC®

Apheresis blood products were collected from 30 donors (phase 1 study: 24 donors; phase 2 study: 6 donors). The collections and study protocol were reviewed and approved by Chesapeake Research Review Inc (Columbia, Maryland) under IRB 0906009, “Development and testing of the MIMIC^®^”. All donors were screened and reported to be in good health. All blood products were received and confirmed to be negative for blood-borne pathogens as detected by standard blood bank screening assays.

Peripheral blood mononuclear cells (PBMCs) were enriched by Ficoll density gradient separation according to standard laboratory procedures [47]. After washing, PBMCs were cryopreserved in dimethyl sulfoxide-containing freezing media for extended storage in liquid nitrogen. Donor PBMCs were chosen at random from our pool for inclusion in this study. In phase 1, 12 “Adult” donors of age <50 years and 12 “Elderly” donors of age ≥65 years were included. All donors for phase 2 were less than 50 years in age.

### MIMIC® Peripheral Tissue Equivalent Assay (phase 1 study)

The PTE construct of the MIMIC® system is designed to replicate the early responses of innate immunity (cytokines and antigen presenting cell activation/maturation) in response to test agents [11, 13, 16, 17]. The MIMIC® PTE module used in this study was built around our published manual technique but automation was used for cell and treatment application and washing steps [13]. Briefly, endothelial cells were grown to a confluent layer atop a collagen matrix (PureCol; Advanced Biomatrix, San Diego, California). Thereafter, donor PBMCs were prepared from frozen stocks and applied to MIMIC® PTE assay wells. After an incubation period, non-migrated cells were washed away leaving only those cells that had transmigrated across the endothelial barrier into the collagen matrix. Over the course of a 48-h incubation period, antigen presenting cells (APCs), primarily differentiating immature dendritic cells, reverse-transmigrate back across the endothelial barrier (Additional file 1: Fig. S1). Test agents including mRNA vaccine were added to the constructs at the indicated concentrations 24 h prior to cell collection. MIMIC®-PTE modules were left untreated or treated with increasing concentrations of the mRNA vaccine (5–50 μg/10^6^ cells), benchmark influenza vaccine (Fluzone® Trivalent 2012–13, FZ, 1:100), or the TLR7/8 agonist R848 (5 μg/ml). 24 human donors were assessed.

The reverse transmigrated cells were harvested after the 48-h incubation period for phenotyping analysis by flow cytometry. The cells were harvested, washed, and labeled for viability with LIVE/DEAD Aqua (Invitrogen, Eugene, Oregon). The cells were then labeled with a multicolor antibody panel specific for cluster of differentiation (CD) 14, human leukocyte antigen-DR, lymphocyte markers (CD3/CD19), and markers of immune cell activation/maturation (CD86, CD40, CCR7, CD25). All antibodies were purchased from eBiosciences (San Diego, California) or BD/Biosciences (San Jose, California). Data was acquired on a BD FORTESSA II flow cytometer (BD/Biosciences) and analyzed using FlowJo software (TreeStar Inc, Ashland, Oregon). Culture supernatants of MIMIC® PTE assays were also analyzed by multiplex bioplex analysis for cytokines and chemokines involved in innate immune cell activation and response [Millipore MILLIPLEX® human cytokine/chemokine kit(s)]. Levels of cytokines were measured in the pg/ml range, allowing for comparison of treated immune cell versus untreated control PTE wells.

### MIMIC® Transwell Peripheral Tissue Equivalent Assay (phase 2 study)

As with the MIMIC® PTE construct, the MIMIC® Transwell PTE construct is also designed to replicate the early processes of innate immunity in response to test agents, albeit in a larger-scale manner [47]. In this system, endothelial cells were grown to a confluent layer atop a transwell membrane. Thereafter, donor PBMCs were prepared from frozen stocks and applied to MIMIC® TW-PTE assay wells. After an incubation period the bucket containing the non-migrated cells was removed leaving only those cells that had transmigrated across the endothelial barrier into the bottom transwell bucket. As in the MIMIC® PTE constructs, these cells were primarily composed of differentiating immature dendritic cells and a small population of leukocytes comprised of B-cells (1–5%) and T-cells (15–20%) and were cultured for 48 h before collection. 24 h prior to collection the mRNA vaccine was added to the TW-PTE modules at 25 μg/10^6^ cells. As a positive control, 5 μg/ml R848 was added to the constructs.

Use of the larger-scale MIMIC® TW-PTE system allowed for the collection of enough cells for RNA isolation and purification for use in full genome microarray analysis, all while retaining the cell populations and innate response profile found in MIMIC® PTE modules.

### Biopsy of mouse injection sites (phase 3 study)

The mRNA vaccine was applied via intradermal injection, distributed to two sites on the backs of C57BL/6 mice. 2 × 50 μl of mRNA vaccine dissolved in Ringer’s lactate solution were injected, for a total amount of 80 μg of mRNA vaccine. Biopsies were collected 6 or 24 h post treatment from the injection site (two approx. 1 cm^2^ pieces per mouse) and the dLN (axillary and brachial, four dLN in total). The time points of analysis were selected so that earlier (6 h) and later (24 h) effects could be measured. Animals treated with buffer served as controls. Untreated mice were used as an additional control to exclude the possibility of unspecific effects induced by the injection of the buffer. The animal protocol (CUR6-12) was approved by the regional council in Tuebingen, Germany.

### RNA samples from MIMIC® TW-PTE (phase 2 full genome microarray analysis)

24 h after treatment, MIMIC® TW-PTE donor samples (n = 18) were collected and the cells were counted. Briefly, following harvest from the PTE at least 1 × 10^6^ immune cells, composed primarily of immature dendritic cells with a small subset of T-cells and B-cells, were lysed in Buffer RLT (QIAGEN) with freshly added 2-mercaptoethanol and stored at −80 °C. After all time points were collected, the samples were thawed, and the RNA isolation proceeded according to the manufacturer’s protocol (QIAGEN). Total RNA sample quality was evaluated by spectrophotometer to determine quantity, protein contamination and organic solvent contamination, and an Agilent 2200 Tapestation was used to check for RNA degradation. Two-round in vitro transcription amplification and labeling was performed starting with 50 ng intact, uncontaminated total RNA per sample, following the Affymetrix protocol. After hybridization on Human U133 Plus 2.0 Arrays for 16 h at 45 °C and 60 r.p.m. in a Hybridization Oven 640 (Affymetrix), slides were washed and stained with a Fluidics Station 450 (Affymetrix). Scanning was performed on a seventh-generation GeneChip Scanner 3000 (Affymetrix), and Affymetrix GCOS software was used to perform image analysis and generate raw intensity data. Initial data quality was assessed by background level, 3′ labeling bias, and pairwise correlation among samples. For this analysis, we used Affymetrix Human Genome U133 Plus 2.0 Array, but instead of using Affymetrix’s sequence clusters to define genes, which is based on the UniGene database build 133, 20 April 2001, gene sequence clusters were based on the updated UniGene build 199, 16 January 2007, to yield a list of 20,078 genes.

### Microarray analysis (phase 2 full genome microarray analysis)

Gene expression data was analyzed using Array Studio (Omicsoft, V7.2). The data was normalized and a MAS5 report was generated for QC assessment. The ArrayStudio (V7.2), Ingenuity Pathway Analysis (IPA, http://www.ingenuity.com) and GeneGo (Thomson Reuters, MetaCore version 6.19, build 65960) packages were used to identify differentially expressed genes (pFDR < 0.05; fold change >1.3 and <−1.3) compared with mock condition.

### Data processing and statistical analysis (phase 2 full genome microarray analysis)

Initial quality control of the microarray signal intensity data was performed using the lumi Bioconductor package [48] in the R programming language. Regression and ANOVA were carried out in R. Further analysis was carried out using ArrayStudio. Array Studio, Array Viewer and Array Server and all other Omicsoft products or service names are registered trademarks or trademarks of Omicsoft Corporation, Research Triangle Park, NC, USA. Statistical analysis was performed using the SAS environment package JMP® (JMP®,Version 10. SAS Institute Inc., Cary, NC, 1989–2007).

### Pathway enrichment and content analysis (phase 2 full genome microarray analysis)

The gene ontology vocabulary used was obtained from the GO Web site (http://www.geneontology.org, 2014 build). Genes that had shown to be significantly modulated by vaccination, as determined by the microarray analysis were further analyzed for pathway enrichment. Briefly, we used ArrayStudio to analyze the microarray data by pairwise scatter analysis and identify significantly differentially regulated genes. The differentially expressed genes were defined in terms of the log2-fold change for treatment over mock. To limit the detection of false positives, the array data was set with thresholds including p values adjusted by the Benjamini and Hochberg false-discovery-rate method with a cutoff of 0.05. Gene lists were analyzed using GenGo MetaCore analysis software (Thompson Reuters), Ingenuity Pathway Analysis software (Ingenuity Systems) and DAVID Ontology (http://www.david.abcc.ncifcrf.gov) to identify significantly associated pathways and generate pathway maps.

### Phase 3 full genome microarray analyses from mouse tissue biopsies

Total RNA was isolated from RNAlater-preserved biopsy tissues with commercially available kits and gene expression analysis was performed by the service provider MFT Tuebingen, Germany. For this purpose 100 ng of total RNA was amplified per array with the Ambion WT expression kit according to the manufacturer instructions and labeled. The samples were then hybridized and stained on the Affymetrix WT Mouse Gene-2.1-ST GeneChip Array using the Affymetrix hybridization, wash, and stain kit. The arrays were scanned with the Affymetrix GCS3000 reader. The raw data were read in the AGCC 3.0 software and converted to intensity values. Further analysis of the data was performed in R 2.15.1 on various Bioconductor packages. Some arrays did not meet the quality control criteria (one skin sample from group 4, one skin sample from group 5 and one dLN sample from group 3 and were excluded from further analysis. Since at least four replicates per condition were still available, the impact on the statistical analysis should be considered as very low.

To identify differentially expressed transcripts the arrays were normalized via RMA (Robust Multichip Average) [49]. All subsequent steps were separated by tissue. For the calculation of differentially expressed transcripts a linear model was created for the comparisons of the mRNA vaccine treated groups and the respective buffer controls. Before fitting the model control probes were removed and a non-specific variance filter was applied to eliminate non informative transcripts. Subsequently, the coefficients of the linear model that describes the expression profile of the corresponding gene were calculated based on the experimental design [50]. The relevant comparisons were defined as a contrast matrix and the F-statistic was calculated for all comparisons, with the standard error moderated through an empirical Bayesian model [51]. Subsequently, to receive a statement about the significance of the comparisons the p value resulting from the F-statistic was determined and corrected via “Benjamini–Hochberg” for multiple testing for all transcripts followed by a decision matrix [52]. Similarly, the strength of the change in expression (M value) was determined. The M value is the log2 of the fold change. Because many transcripts were differentially regulated only transcripts with a corrected p value less than 0.01 and a log2 fold change greater than 0.9 (fold change greater than 1.87) were taken into account for the subsequent analysis.

### Statistical analysis

All statistical analyses and graphics were prepared using GraphPad InStat version 46.00 (GraphPad Software Inc, San Diego, California). One-way analysis of variance (ANOVA) and Bonferroni posttest analyses were employed to determine statistical significance; p values <0.05 were considered statistically significant.

## Results

### Dose-driven innate response to mRNA vaccine stimulation in human MIMIC®-Peripheral Tissue Equivalent Assay

The purpose of the first phase of this study was to evaluate the immune-stimulatory potential of different concentrations of mRNA vaccine in humans. Human cell-based MIMIC®-PTE modules were used and were either left untreated or were treated with benchmark influenza vaccine (Fluzone®, 2012–13), the TLR7/8 agonist R848, or 6 increasing concentrations of mRNA vaccine (5–50 μg/10^6^ cells). 24 h after the application of treatment human immune cells were harvested from the modules and analyzed for phenotypic markers associated with activation using conventional flow cytometry. Only the reverse-transmigrated cells (those recovered from the media above the endothelial cell layer) were included in this evaluation (Additional file 1: Fig. S1), with the immune cell populations typically comprised of 80–90% APCs, 10–15% CD3+ T-cells, and 1–5% CD19+ B-cells. Culture supernatant was also collected and analyzed by multiplex bioplex analysis for cytokines and chemokines involved in innate immune cell activation and response. Levels of cytokines were measured in the pg/ml range, allowing for comparison of treated versus untreated MIMIC®-PTE modules.

**Fig. 1:**
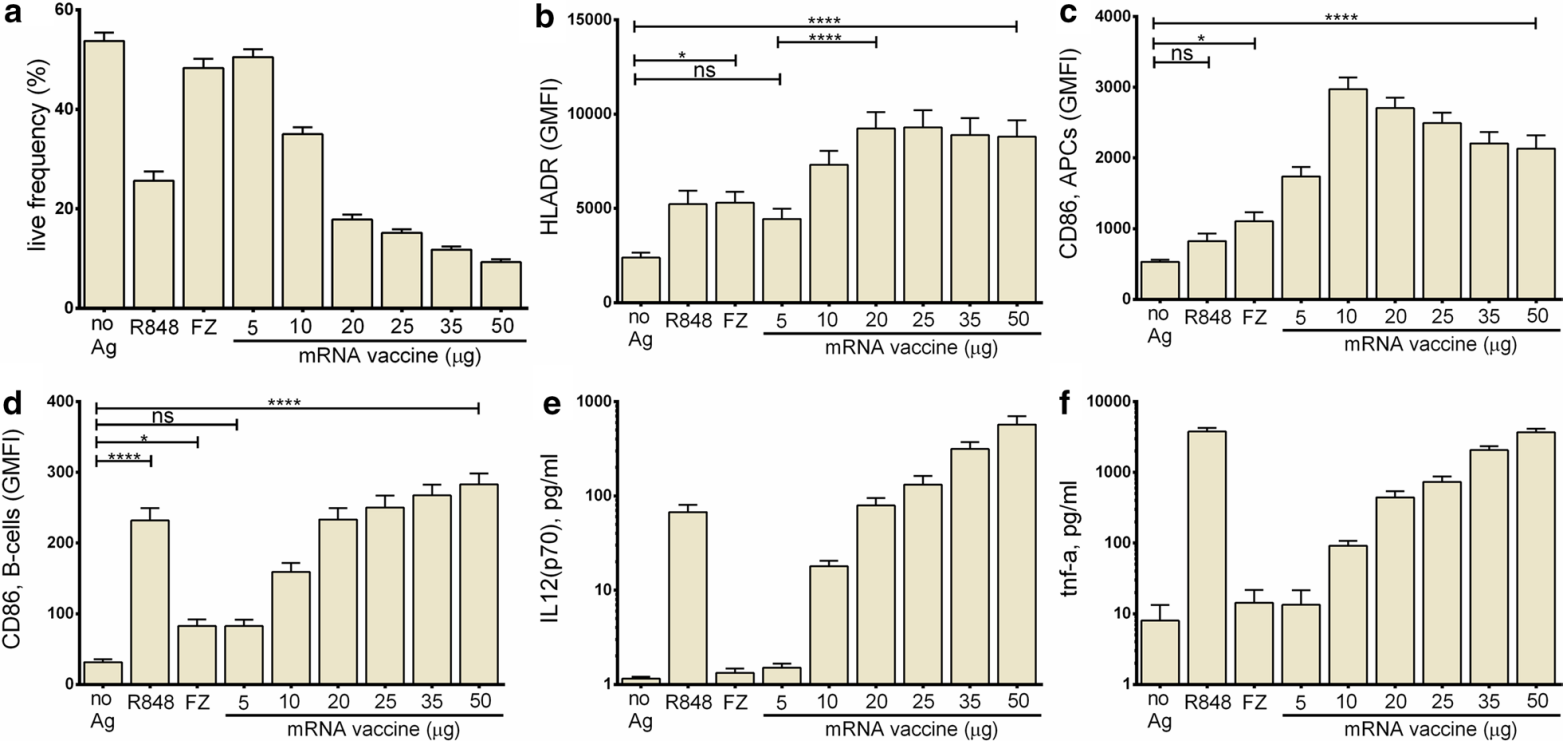
Dose-driven innate response in MIMIC®-PTE. Dose-driven innate response in MIMIC®-PTE immune cell populations to the mRNA vaccine, response that is similar to that of the TLR 7/8 agonist resiquimod (R848, 5 μg/ml). The benchmark influenza vaccine Fluzone (FZ) was also evaluated. **a** Live (live-dead Aqua negative) cell population, as percentage of all singlet cells. Treatment with R848 and higher mRNA vaccine doses drives decreased cell viability and recovery. **b** Dose-dependent maturation effect from the mRNA vaccine in APCs, as demonstrated by up-regulation of HLA-DR. **c** Dose-driven activation of APCs, as demonstrated by up-regulation of CD86. **d** Activation of B-cells, as demonstrated by up-regulation of CD86. **e**, **f** cytokine production in response to TLR7/8 stimulus (R848, 5 μg/ml) and the mRNA vaccine. The mRNA vaccine was dosed at the µg concentration listed per million cells (ex. 5 μg/10^6^ cells). Mean ± SEM are shown for n = 24 subjects examined in MIMIC® modules. Statistical analysis was performed with GraphPad Prism software (version 6.04) using Dunnett’s multiple comparisons test. p value indicators ns, *, and **** refer to “no significant correlation”, p < 0.05, p < 0.0001, respectively

Cell viability assessments were performed on reverse transmigrated cells, including both APC and leukocyte sub-populations. Figure 1a shows the impact of the various treatments on cell viability, activation and cytokine production. There was a dose dependent reduction in cell recovery following treatment with mRNA vaccine, an effect that was most pronounced in the APC cell population (Additional file 2: Fig. S2). Over the dose range examined (5–50 μg/10^6^ cells) the number of HLA-DR+ cells recovered dropped substantially while leukocyte numbers dropped only slightly (Additional file 2: Fig. S2A, B), indicating greater sensitivity of HLA-DR+ APCs for stimulation following mRNA uptake. The impact of treatment with a TLR 7/8 agonist, R848 at 5 μg/ml, also caused a decrease in live cell recovery in the APC population that was equivalent to the highest dose of mRNA vaccine.

Antigen presenting cells respond vigorously to a number of various stimuli such as TLR agonists, antigen, or virus [7, 8, 11, 13, 33, 35, 53]. Mature activated APCs are characterized by expression of markers such as CD14^low^/CCR7^high^/HLA-DR^high^/CD40^high^/CD80^high^/CD86^high^. Reverse-transmigrated cells generated in the MIMIC®-PTE were characterized using flow cytometry to analyze the effect on phenotypic markers of activation of the treatments on APC maturation and activation. As a positive control the TLR 7/8 agonist R848, known to be a highly potent innate stimulus, was shown to trigger APC maturation as evidenced by the down-regulation of CD14 and the up-regulation of HLA-DR (Fig. 1b; Additional file 3: Fig. S3). The mRNA vaccine caused a dose-dependent maturation effect in APCs as evidenced by the up-regulation of HLA-DR (expression level as measured by geometric mean fluorescence intensity, GMFI) an effect that plateaued at the 20 μg/10^6^ cells dose.

Following reverse transmigration in the MIMIC®-PTE module immune cells can potentially be “activated” by innate immune stimuli such as TLR signaling [35, 53]. In APCs and leukocytes a number of surface markers are indicative of the cell’s activation status. These include CD25, CCR7, CD40, and the co-stimulatory molecule CD86. PTE-derived APCs and B-cells were activated by both R848 and mRNA vaccine in a dose-dependent manner. The expression of the co-stimulatory molecule CD86 was greatly enhanced at higher doses of mRNA vaccine in the B cell population and at intermediate doses in the APCs, with maximal expression of CD86 at a dose of 10 μg/10^6^ cells and declining slightly at higher doses (Fig. 1c, d). Expression of CCR7, CD25, and CD40 were also up-regulated by treatment with both compounds (APC profiles shown in Additional file 4: Fig. S4) but were most strongly up-regulated at higher doses of mRNA vaccine.

The mRNA vaccine’s ability to activate MIMIC®-PTE cells to produce cytokines and chemokines with known immune or inflammatory properties was evaluated using the supernatants collected following stimulation. Cytokine production was evaluated at doses ranging from 5 to 50 μg/10^6^ cells and compared to the levels generated following stimulation with the TLR 7/8 agonist R848 at 5 μg/ml. Sentinel markers for innate immune cell activation by TLR ligands include IL-12(p70) and TNF-α (Fig. 1e, f) as well as IFN-α, IL-12(p40), and IL-6 (Additional file 5: Fig. S5). There was a clear dose-dependent increase in the production of these cytokines by mRNA vaccine. Levels of each cytokine also increased after treatment of PTE constructs with R848, indicative of activation of similar signaling pathways to those seen for the mRNA vaccine.

### Shared activation profile from mRNA vaccine and R848 stimulation in human subjects (MIMIC®)

**Fig. 2:**
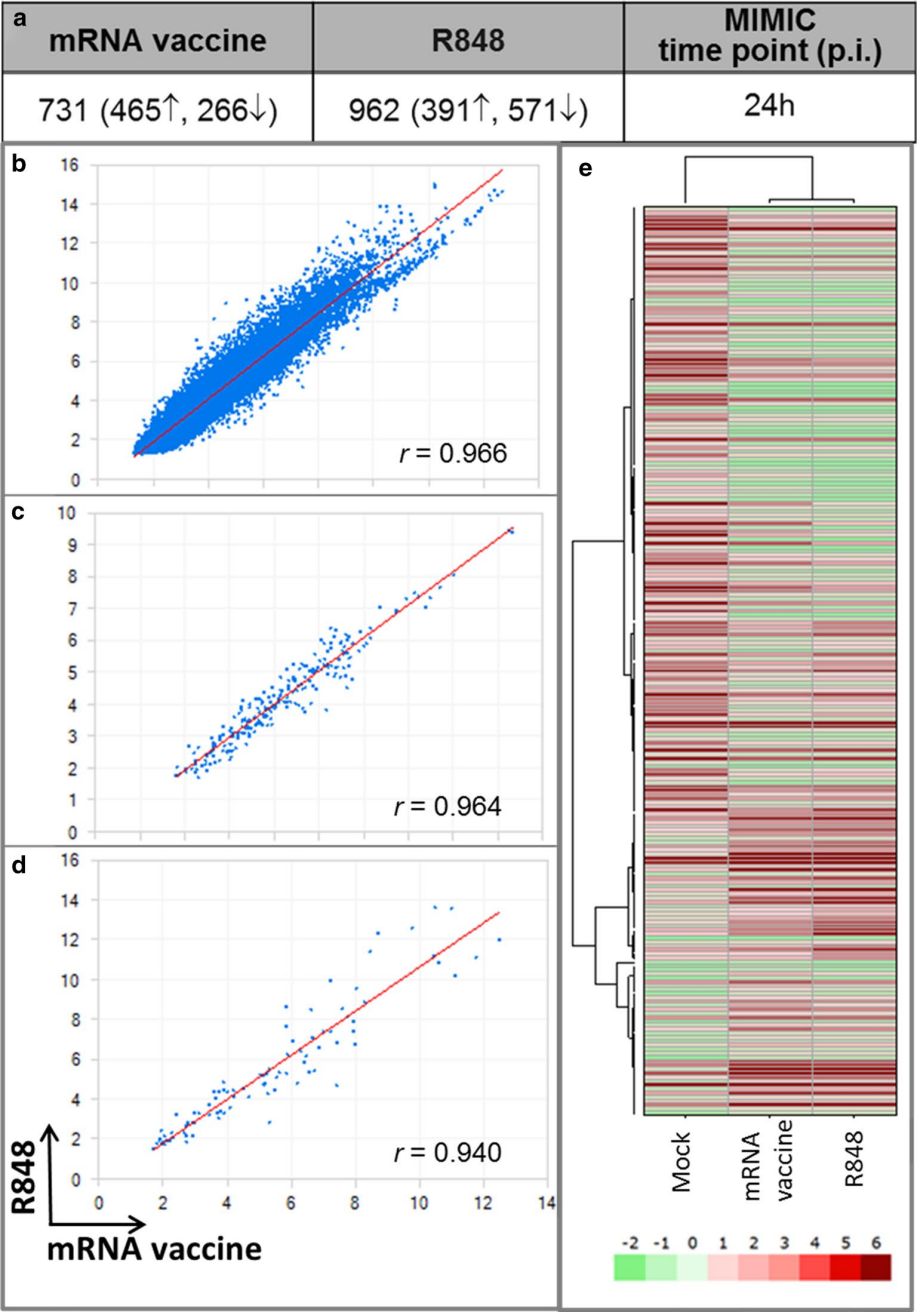
Innate response in MIMIC®-PTE immune cell populations. **a** Number of differentially regulated transcripts relative to the respective no treatment control. Number of up- or down-regulated genes is stated in *parentheses*, *arrows* indicate up-regulated (↑) or down-regulated (↓) genes. Transcriptional response to the mRNA vaccine (RNActive®) showed strong correlation to the TLR 7/8 agonist R848. Correlation between the mRNA vaccine and R848 (correlation plot) in (**b**) all 50,000+ gene transcripts (whole transcriptome), **c** all differentially expressed genes and **d** in the immune-related gene subset that includes the following gene subsets: chemokines, cytokines, antigen presentation, mannose receptors, TLRs, CLRs, MHC class 1 and 2, and other miscellaneous immune-related genes. The mRNA vaccine and R848 trigger comparable transcriptional changes, with Pearson’s correlation coefficients above 0.94. **e** Hierarchical clustering of significant differentially expressed genes in MIMIC®-PTE samples after treatment with no antigen (mock), mRNA vaccine, or R848 treatment. Common up-regulation and down-regulation of genes was evident in samples treated with mRNA vaccine and R848. Number of donors in each group = 6

**Fig. 3:**
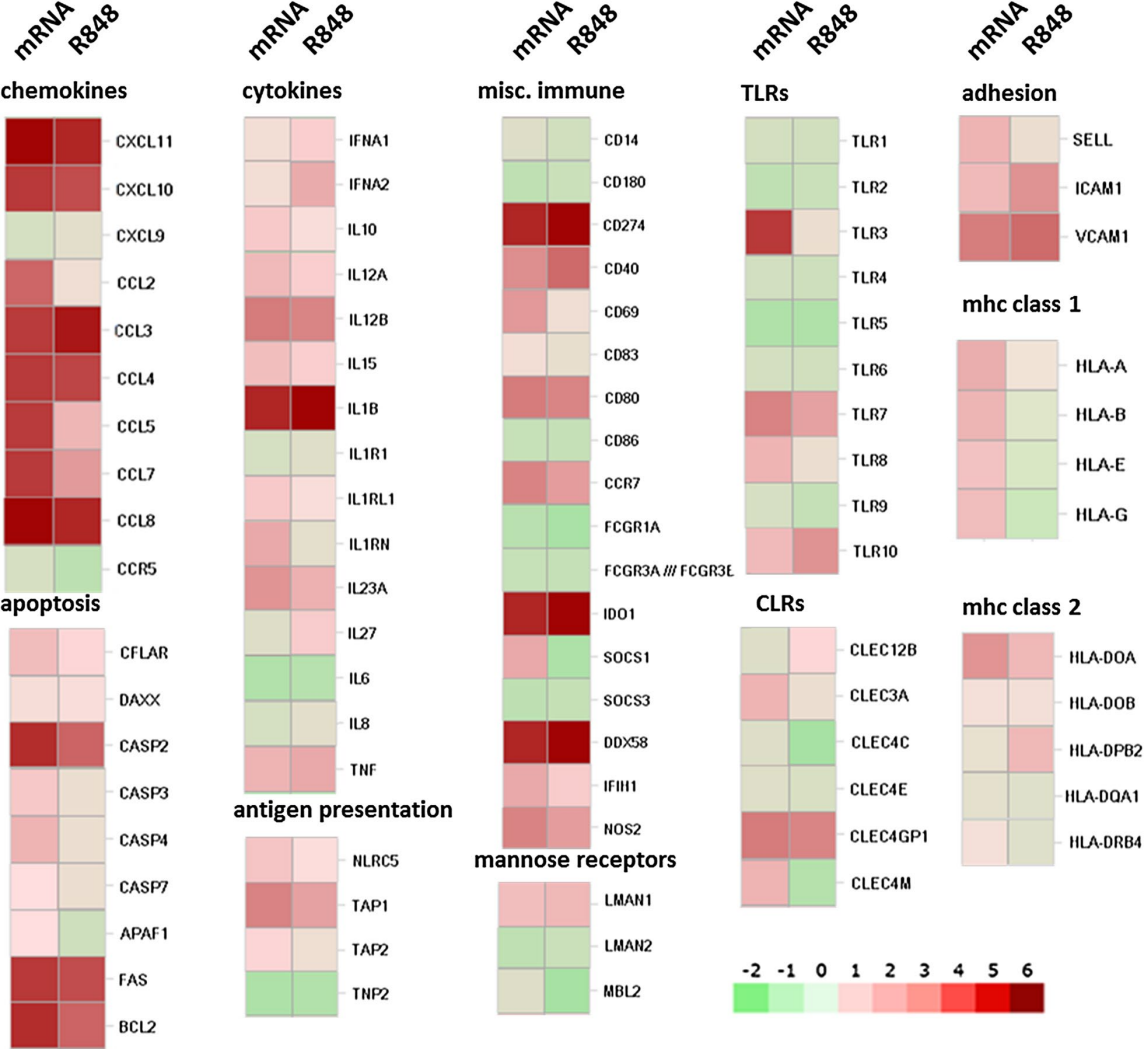
Administration of the mRNA vaccine induces multiple genes of innate immunity in MIMIC®-PTE immune cell populations. Transcriptional response to the mRNA vaccine is compared with R848. The 86 genes shown were taken from the subset of genes designated as immune-related. The heat map represents normalized mean expression values depicted as log2 fold change relative to the respective buffer control. Transcripts were grouped by their immune function. Number of donors in each group = 6

A genome-wide transcriptome analysis has been shown to be useful to identify gene signatures which predict immunogenicity of a number of vaccines in humans [22]. In the second phase of this study, we used the Affymetrix gene chip U133A+ to evaluate activation pathways in MIMIC® TW-PTE derived innate immune cells, primarily composed of differentiating immature dendritic cells and a small population of leukocytes comprised of B-cells (1–5%) and T-cells (15–20%). RNA was extracted 24 h after treatment with the mRNA vaccine or R848 control, purified, and analyzed. Gene expression analysis was performed on both mRNA and R848-treated MIMIC® Transwell-PTE modules, with resulting expression values normalized versus untreated “no antigen” control modules. As shown in Fig. 2a numerous transcripts were differentially regulated following both treatments and the transcriptional response was strongly correlative between the two treatments (Fig. 2b–d). This correlation was evident when evaluating the entire transcriptome (Fig. 2b), all differentially expressed genes (Fig. 2c), and an immune-related gene subset (Fig. 2d) that includes the following gene categories: chemokines, cytokines, antigen presentation, mannose receptors, TLRs, CLRs, major histocompatibility complex class 1 and 2 (MHC), and other miscellaneous immune-related genes. The hierarchical clustering of these significant differentially expressed genes is shown in Fig. 2e. The immune-related gene subset that showed correlation in Fig. 2d is listed on a gene by gene basis in Fig. 3. The majority of genes listed display common patterns of regulation.

Of particular interest in this transcriptional analysis was the identification of PRR’s up-regulated after mRNA vaccine stimulation and the identification of immune-related pathways that were most significantly activated. Genes for RLRs [*ddx58* (RIG-1) and *ifih1* (MDA-5)], TLRs (*tlr3*, *tlr7*, and *tlr8*), and CLRs (*clec4gp1*, *clec2d*, *cledl1*) were all significantly activated by the mRNA vaccine (Fig. 3), generating a response profile characteristic of dsRNA or ssRNA-receptor binding. R848, specifically indicated as an agonist for TLR7/8, did not strongly induce TLR3 where the mRNA vaccine did. This activation of TLR3 and RLRs is suggestive of double-stranded structures in the mRNA vaccine. Additionally, this response profile indicated that the mRNA vaccine enters the cell through a mechanism that leads to activation of receptors in endosomal compartment and the cytoplasm.

MHC class I and II gene activity increased in response to treatment with mRNA vaccine, indicating a maturation of the APC cell population (Figs. 1b, 3). Down-regulation of *cd14* transcripts correlated well with this shift of phenotype. These results align with the phenotyping results indicating APC maturation as evidenced by the shift to a CD14^low^/HLA-DR^high^ phenotype (Additional file 3: Fig. S3). The phenotyping results indicated that the co-stimulatory surface marker CD86 increased substantially in the MIMIC®-PTE population harvested and analyzed after 24 h (Fig. 1c). However, at this time point *cd86* gene activity dropped in the mRNA vaccine treated cells versus no antigen control.

Chemokine and cytokine patterns of induction following treatment with the mRNA vaccine were similar as evidenced by the increased expression of several markers of innate immune cell activation by TLR ligands including IL-12(p40), IL-12(p70), IFN-α, and TNF-α (Fig. 1e, f; Additional file 5: Fig. S5). Enhanced expression of cytokines and chemokines correlated with increased cytokine/chemokine related gene activity (Fig. 3). An exception to this was the lack of increased gene activity corresponding to increased levels of IL-6 and IL-8. In the MIMIC® TW-PTE increases in IL-6 and IL-8 are driven primarily by the endothelial cell population (data not shown). Because the transcriptome analysis was performed only on the immune cell population which was physically separate from the endothelial cells, up-regulation of *il*-*6* and *il*-*8* was not expected to be observed. One glaring difference between the mRNA vaccine and R848 was seen in the induction of *il*-*27*. *il*-*27* induction was down-regulated from the mRNA vaccine and significantly up-regulated by R848. This cytokine is reported to promote CD4+ T cell differentiation to the T helper (Th) 1 lineage and suppresses Th2 and Th17 differentiation and to promote type 1 regulatory (Tr1) which produce IL-10 [54, 55].

The most significant human immune pathways induced following stimulation with the mRNA vaccine include those associated with the TLR, IL-1, and JNK (c-jun N-terminal kinases) pathways (Additional file 6: Fig. S6). IL-1 and JNK are part of the NF-κB and mitogen-activated protein kinase (MAP kinase) signaling pathways, activation pathways into which PRRs converge to transduce signals.

### Multiple innate-associated genes are induced in mice following ID administration of mRNA vaccine

**Fig. 4:**
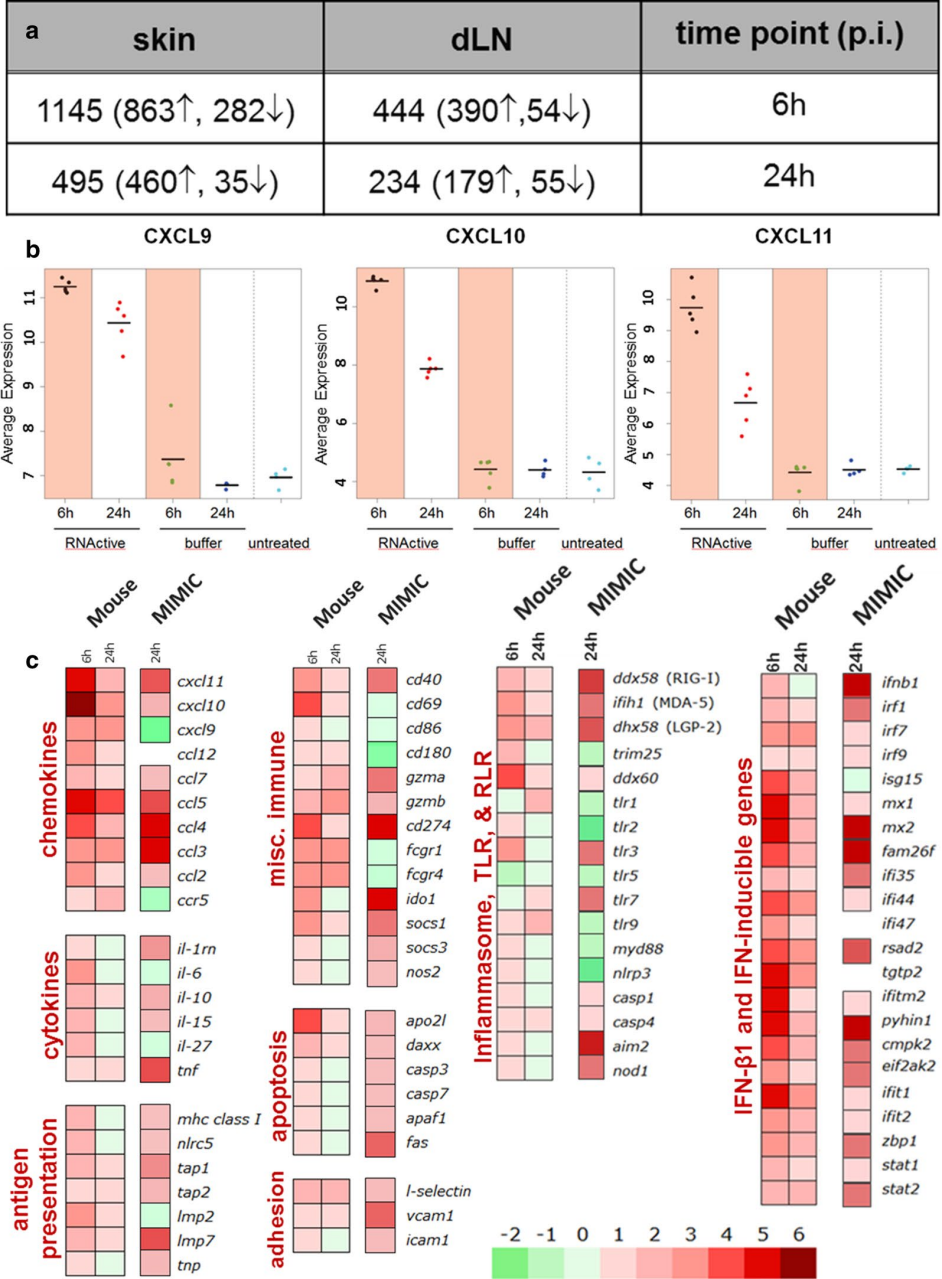
mRNA vaccine induced multiple genes of innate immunity at the injection site, in the dLN in mice, and in the human MIMIC®. C57BL/6 mice were treated via the ID route with the indicated amount of mRNA vaccine distributed to two injection sites on the back of the mice. **a** Number of differentially regulated transcripts relative to the respective buffer control. Number of up- or down-regulated genes is stated in *parentheses*, *arrows* indicate up-regulated (↑) or down-regulated (↓) genes. Administration of mRNA vaccine increased the gene expression in the skin 6 h post administration of mRNA vaccine RNA was isolated from RNAlater-preserved skin biopsies collected 6 or 24 h post ID application of the mRNA vaccine. **b** Increase of the gene expression of CXCR3-ligands in the skin 6 h post administration of the mRNA vaccine. The *dots* indicate the signal intensity of each sample, *cross-bar* corresponds to the mean value. The groups compared with each other are highlighted. **c** RNA was isolated from RNAlater-preserved skin biopsies collected 6 or 24 h post ID application of the mRNA vaccine. The Heat map represents normalized mean expression values depicted as log2-fold change relative to the respective buffer control. Transcripts were grouped by their immune function. Heat maps representing transcriptional changes in MIMIC® immune cell populations collected 24 h after treatment with mRNA vaccine were included for comparison

The third phase of this study was performed in C57BL/6 mice to assess the mechanisms of self-adjuvantation of mRNA vaccines in vivo. The mRNA vaccine was applied via intradermal injection, distributed evenly between two sites on the backs of C57BL/6 mice. Biopsies were collected 6 or 24 h post-treatment from the injection site (two approx. 1 cm^2^ pieces per mouse) and the dLN (axiliary and brachial, four dLN in total) were collected. Both cellular and molecular sensors at the injection site and in the draining lymph nodes were evaluated for phenotype, chemokine production, and gene expression patterns. As shown in Fig. 4a numerous transcripts (>1000) were differentially regulated in the skin early following mRNA vaccine treatment. Changes in the gene expression after treatment were also detected in the dLN, however the number of differentially regulated transcripts was clearly lower here than observed in the skin samples.

Administration of the mRNA vaccine led to significant transient induction of distinct chemokines, cytokines and activation markers of immune cells locally at the injection site. Among the chemokines the CXC chemokine receptor (CXCR) 3-ligands CXCL9, CXCL10 and CXCL11 whose pleiotropic functions include stimulation of monocytes/macrophages, T cells, NK cells, and dendritic cell migration showed the most pronounced up-regulation (Fig. 4b, c left panel). The gene expression level of CCL5, a chemokine that selectively supports the migration of CD4-expressing monocytes and T lymphocytes was also strongly elevated. This coincided with up-regulated gene expression of CCR5, the receptor of CCL5 (Fig. 4c, left panel). Pro-inflammatory cytokines IL-6, TNF-α as well as IL-27, a heterodimeric cytokine belonging to the IL-12 family were increased early upon vaccine administration. In addition, elevated gene expression of CD69, CD40 and CD86 was detected early upon mRNA vaccine treatment indicating specific activation of immune cells in the skin (Fig. 4c). CD86 up-regulation suggests activation of antigen presenting cells. Of note, the key regulator of MHC class I-dependent immune responses NLRC5 along with several components of the MHC class I antigen processing and presentation pathway showed elevated gene expression in the skin early upon mRNA vaccine injection (Fig. 4c, left panel).

### ID mRNA vaccine injection in mice induces multiple genes of innate immunity in the dLN

**Fig. 5:**
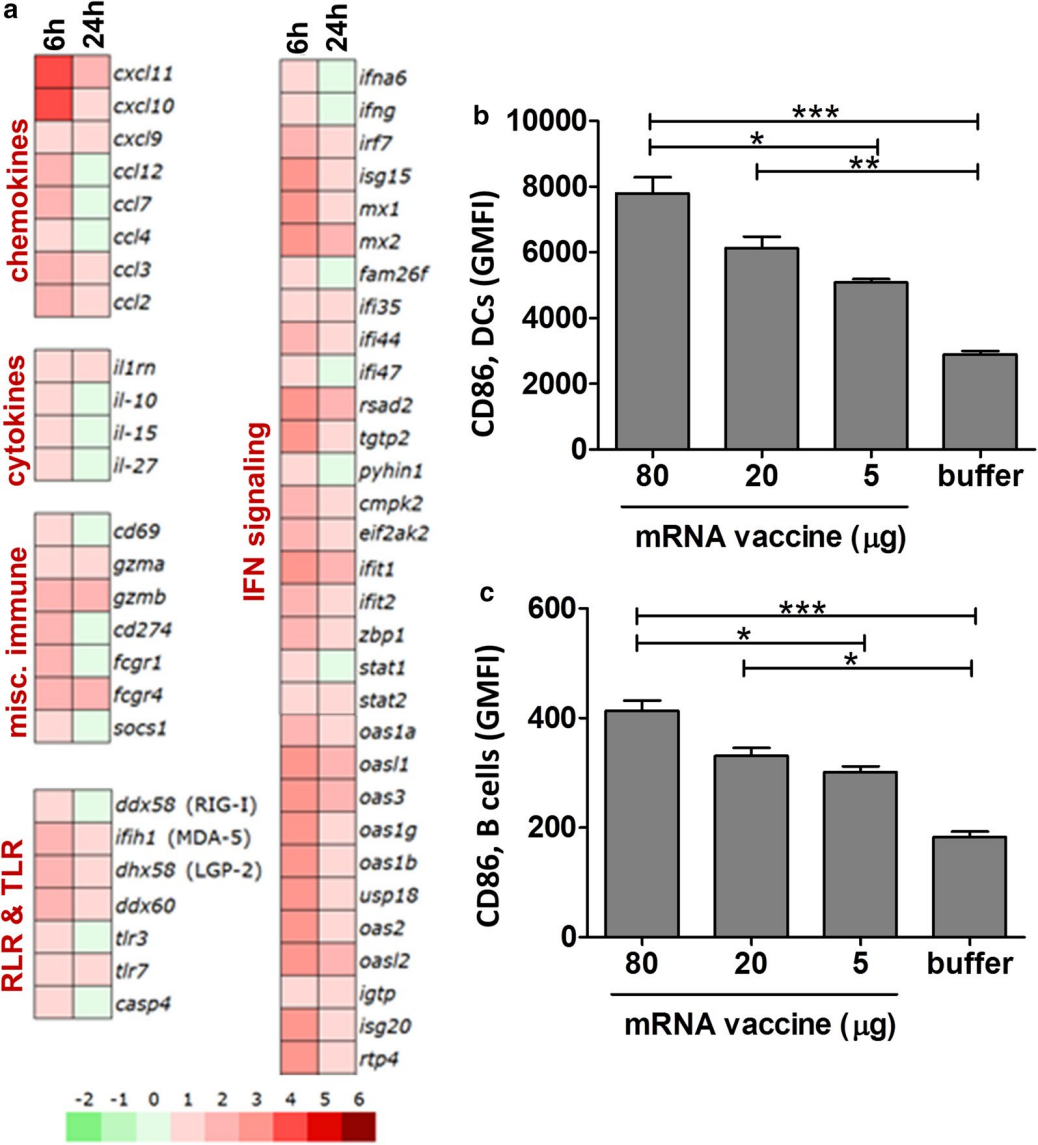
mRNA vaccine induced multiple genes of innate immunity in the dLN. RNA was isolated from RNAlater-preserved LN biopsies (axillar and brachial) collected 6 or 24 h post ID application of mRNA vaccine. Whole genome expression analysis was performed using Affymetrix WT Mouse Gene-2.1-ST GeneChip Array. **a** Heat map represents normalized mean expression values depicted as log2 fold change relative to the respective buffer control. Transcripts were grouped by their immune function. **b**, **c** dLN (axillar, brachial and inguinal) were harvested 24 h post treatment and single cell suspensions were analyzed using polychromatic flow cytometry gating on live cells by followed by staining for markers of (**b**) migratory dendritic cells (CD11c^+^MHCII^high^ cells) and (**c**) B cells (B220^+^CD19^+^ cells). The surface expression of CD86 on dendritic cells and B cells was determined as geometric mean fluorescence intensity (GMFI). Mean ± SEM are shown for n = 8 mice per group. Statistical analysis was performed with GraphPad Prism software (version 5.04) using Kruskal–Wallis test followed by Dunns multiple comparison test. p value indicators ***, **, * refer to p < 0.05, p < 0.01 and p < 0.001, respectively

In general, a similar profile of the changes in gene expression as described for the skin was observed in the dLN. However, compared to the skin, the intensity of the up-regulation of the expression of certain genes was slightly weaker and several factors such as CCL5, IL- 6, TNF-α, apoptosis-related genes and some inflammasome components were not elevated in the dLN (Fig. 5a). IFN-α6 and IFN-γ which were not detected in the skin showed augmented gene expression in the dLN following mRNA vaccine treatment (Fig. 5a, right panel). Single cell suspensions from the dLNs were analyzed using polychromatic flow cytometry gating on live cells followed by staining for markers of immune cell activation. Up-regulation of CD86 was detected in both migratory dendritic cells and B cells (Fig. 5b, c). Administration of the mRNA vaccine induced dose-dependent activation of dendritic cells and B cells in the dLN 24 h post treatment.

### Correlation and differences in innate response to mRNA vaccine between human (MIMIC®) and mice

**Fig. 6:**
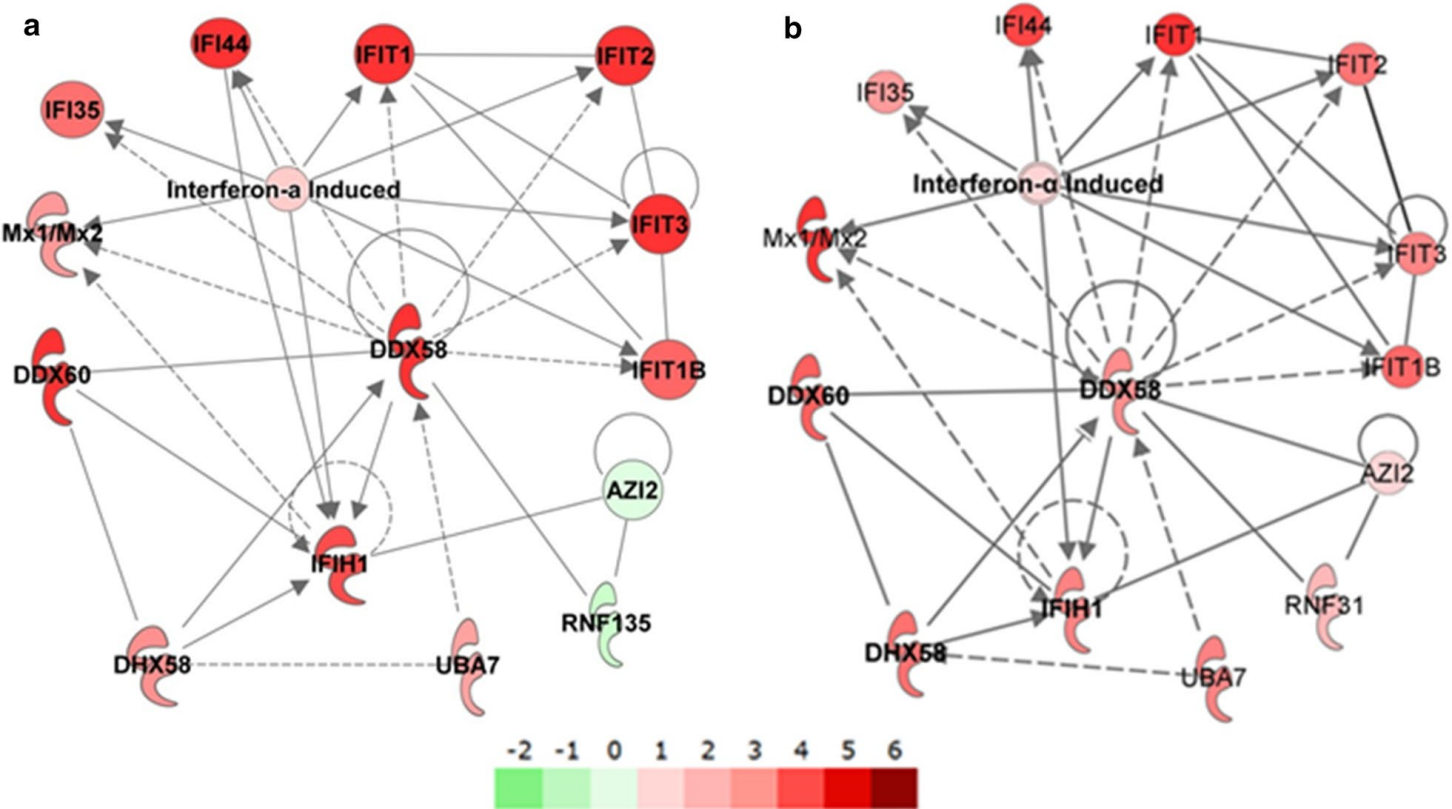
Analysis of RIG-I (encoded by Ddx58 gene) signaling network after mRNA vaccine treatment in humans (MIMIC®) and mice. **a** RNA generated from MIMIC®-PTE immune cell populations 24 h after modules were treated with the mRNA vaccine. **b** The mRNA vaccine was injected into mice, and 6 h post administration RNA was isolated from RNALater-preserved skin biopsies. Up-regulated genes are shown in *red*, *solid lines* indicate direct interactions, *dashed lines* indirect effects, *arrow heads* indicate causality

Analyses of the receptors that play a role in the recognition of the mRNA vaccine by the innate immune system revealed that cytoplasmic RNA sensors of the retinoic acid-inducible gene (RIG)-I-like receptor family such as RIG-I and MDA-5 as well as the positive regulator of RIG-I- and MDA-5-mediated response, LGP-2 were elevated early upon mRNA vaccine administration in the injection site and in the human MIMIC®-PTE (Fig. 4c). Additionally, the gene expression of the RING-finger E3 ubiquitin ligase TRIM25 which induces the Lys-linked ubiquitination of RIG-I and is therefore crucial for RIG-I-mediated activity was also up-regulated early in the injection site. This contrasts with down-regulation of this gene in the MIMIC® that may be due to the evaluation of these samples 24 h after treatment or species-specific differences in mRNA vaccine-induced cell activation. Data from the murine skin samples supports the possibility of a temporal parameter since a similar down-regulation was observed in the mouse injection site samples 24 h after treatment. The helicase Ddx60 that promotes the binding of RIG-I to dsRNA showed increased gene expression in both the MIMIC® and the mouse injection site. Taken together, our analyses indicate that RIG-I-like receptor-mediated signaling network participates in the sensing of the administered mRNA vaccine in the skin of the mouse and the human MIMIC® (Fig. 6), with common up-regulation of genes demonstrated in both species.

Besides the RLRs, Toll-like receptors and NOD-like receptors (NLR) also showed differential gene expression upon mRNA vaccine treatment. The endosomal RNA receptors TLR3 and TLR7 were up-regulated with TLR3 showing enhanced expression levels early in the injection site (6 h) and in the human MIMIC®. While TLR7 increases were detected in the MIMIC® sample, TLR7 increase was only detected in the late (24 h) sample following mRNA vaccine treatment in the mouse (Fig. 4c). The up-regulation of TLR9 in the injection site of the mouse, which is not a RNA-sensor but recognizes specific unmethylated CpG motifs prevalent in microbial genomic DNA can most probably be explained as an unspecific bystander effect mediated by the local pro-inflammatory milieu induced by the mRNA vaccine. Notably, the gene expression of several components of the inflammasome signaling pathway such as AIM2, NOD-1, caspase-1 and caspase-4 was also increased 6 h after mRNA vaccine injection in the mouse and in human MIMIC®-PTE modules.

**Fig. 7:**
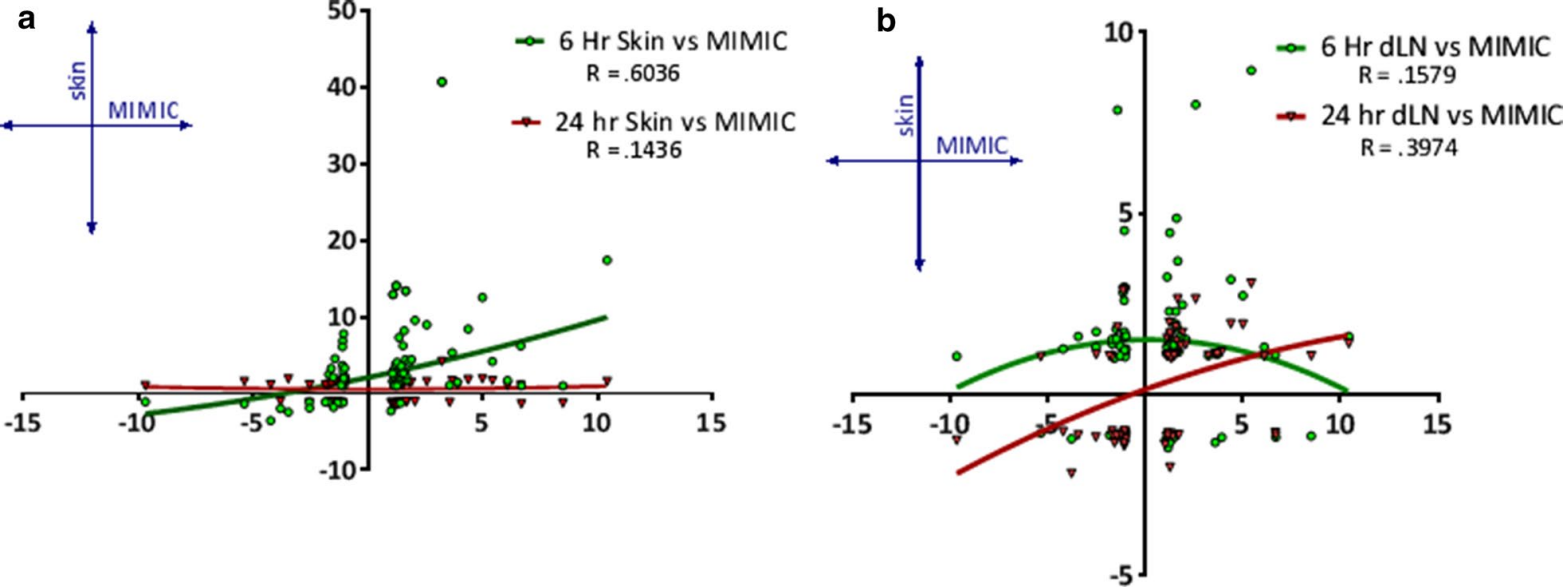
Correlation plot for human MIMIC® and murine response in the immune-related gene subsets listed in Figs. 3 and 4. **a** MIMIC® versus 6 and 24 h injection site transcripts, **b** MIMIC® versus 6 and 24 h dLN transcripts. Response was most correlative between MIMIC®-PTE and 6 h injection site innate response, as evaluated with second order polynomial fit (quadratic). R values are listed

Further evaluation was performed on the genes of innate immunity listed in Fig. 3 to correlate transcriptional response between the human (MIMIC®-PTE) and the mouse. In the mouse, four data sets were evaluated: 6 and 24 h injection site (skin), 6 and 24 h dLN. Transcriptional response to the mRNA vaccine showed a moderately positive correlation in innate response between human MIMIC® and mouse injection site 6 h after injection, with an R value of 0.6036 (Fig. 7a). This correlation is lost between MIMIC and the 24 h injection site sample. Between the MIMIC® and the dLN correlation in transcriptional response improved between 6 and 24 h, but overall limited correlation is observed Fig. 7b. This loss of correlation from the 6 and 24 h skin biopsy and MIMIC® with simultaneous improvement in correlation from the 6 and 24 h dLN samples and MIMIC® could indicate migration of the activated innate immune population from the site of injection to the lymph node. Species-specific differences and limitations to the in vitro model could also account for less than perfect correlation between the samples.

## Discussion

In this study, we evaluated the innate immunostimulatory potential of an mRNA vaccine encoding influenza A hemagglutinin, a vaccine that previously demonstrated protective immunity to influenza A virus infections in mice and pigs [45]. This evaluation was performed in an in vitro model of human innate immunity and in vivo in mice after intradermal injection. To evaluate the self-adjuvant properties of this novel vaccine a translational approach was undertaken to correlate phenotypic and cytokine/chemokine responses in immune cell populations to transcriptional responses in those same cells. In the human MIMIC® strong correlation was demonstrated in phenotypic, cytokine, and transcriptional response between the TLR7/8 agonist R848 and the mRNA vaccine indicating that at least a part of the signaling involved these TLR receptors. However, there were some noteworthy differences in transcript up-regulation. Both *il*-*27* and *il*-*8* were down-regulated following treatment with mRNA vaccine and up-regulated by R848. *ddx58* (RIG-1), *ifih1* (MDA-5), and *tlr3* were all up-regulated by the mRNA vaccine to a much greater degree than from R848. These differences support the notion that the vaccine does not act solely through stimulation of the TLR7/8 pathway. The adjuvant effect of mRNA vaccine in humans (MIMIC®) and mice acts through similar cellular RNA sensors found in endosomal compartments as well as within the cytoplasm of immune cells. Transcriptional analysis demonstrated up-regulation of TLR3, 7, and 8 in humans, TLR 3 and 7 in mice, and RLRs such as RIG-I, MDA-5, and inflammasome components in both species. Sixty-five of the 81 “innate” immune-related genes identified in this study demonstrated correlative transcriptional regulation in human MIMIC® modules and at the injection site in mice 6 h after treatment. The most significant immune pathways induced in response to the mRNA vaccine include the TLR, IL-1, and JNK pathways. Results from the phenotypic analysis of immune cell populations and cytokine/chemokine levels of treated human MIMIC® modules and ID injected mice confirmed the immunostimulatory capacity of the mRNA vaccine. Phenotyping revealed immune cell maturation and activation of APCs and B-cells. Cytokine/chemokine analysis indicated production of factors in both systems that could attract and activate key players of the innate and adaptive immune system.

Similarities and differences have been demonstrated between mice and humans in immune system development, activation, and response to challenge [2–6]. The relevance of any study in mice into the effects that immunostimulatory agents and adjuvants to the human response depends upon whether those stimuli target pathways that are conserved or convergent between mice and humans and whether it is realistic to single out particular genes for analysis. In this study the focus was on predicting the innate response in humans following treatment with an mRNA vaccine using two model systems, a human in vitro model and a murine model. The mRNA vaccine largely drove consistent responses in the two despite some species-specific differences in cell populations, differences in RNA sensors between the species, and fundamental differences between in vitro models and in vivo testing. When comparing mice and humans, some differences have been noted in the principle ssRNA sensors present in immune cell populations. In mice, dendritic cells typically express TLR7 whereas in humans TLR8 is present on myeloid-derived DCs (which predominate in our system) while TLR7 can be found on plasmacytoid DCs and B-cells (subsets also found in the MIMIC®) [31, 32, 56]. Based on the transcriptional profile measured in both humans and mice, genes for RLRs [*ddx58* (RIG-1) and *ifih1* (MDA-5)], TLRs (*tlr3*, *tlr7*, and *tlr8*-*human only*), and CLRs (*clec4gp1*, *clec2d*, *cledl1*) were all significantly up-regulated by the mRNA vaccine. The up-regulation of TLR8 and TLR7 points to the involvement of both mDCs and pDCs in the innate response to the mRNA vaccine in humans. The induced production of IFNα from the mRNA vaccine suggests that pDCs present in MIMIC® were activated. TLR3 and RLRs were activated in the mouse and the human MIMIC ® indicating the probability of double-stranded structure in the mRNA vaccine that effectively amplifies the adjuvant effect of the vaccine. These endosomal and cytoplasmic sensors of dsRNA do not typically respond to R848 and while gene families for both are up-regulated slightly by R848 the mRNA vaccine triggered much greater up-regulation of these sensors and activation of relevant downstream pathways. This overlap of results between the mouse and human models highlight the relevance of each for studying a subset of conserved gene families when evaluating the adjuvant effects of this vaccine. The models complement each other to highlight receptors that are conserved between species and known to generate innate responses following challenge with RNA. In some cases, however, transcriptional differences were observed between the mouse and human, likely due to different patterns of cellular sensors on innate immune cell populations. The gene for *il*-*27* is up-regulated early in the injection site but is down-regulated late in the injection site and in the MIMIC® possibly due to differences in the pattern of response between the mouse and human, specifically in TLR7/8 activation. This is supported by the up-regulation of *il*-*27* in the MIMIC® from R848 which activates TLR7 but down-regulation from the mRNA vaccine which appears to activate TLR8 in mDCs.

In addition to consistent transcriptional responses between the mice and the human subjects evaluated in this study, mRNA vaccination resulted in phenotypic and cytokine/chemokine responses that were similar between the two species and reflected the transcriptional profile. Elevated expression of genes for CD69 and CD40 was detected early upon mRNA vaccine injection in mice indicating specific activation of immune cells in the skin. Increased surface expression of CD86 was measured in human MIMIC®-PTE APCs and B-cells and also in migratory dendritic cells and B-cells in the draining lymph nodes of mice, all suggesting activation of antigen presenting cells. However in the PTE module at this time point *cd86* gene activity dropped in the mRNA vaccine treated cells versus no antigen control. This decline may have been due to an initial burst in *cd86* activity followed by a subsequent decline since the dose used in the transwell experiments (25 μg) was higher than that showing optimal immune cell activation on the APC population from the PTE (10 μg). Another possibility to explain the discrepancy between phenotype and gene expression of CD86 is that there exists an intracellular reservoir of CD86 in dendritic cells [57]. These intracellular reservoirs can cycle CD86 to the cellular membrane rapidly in response to cell activation. mRNA vaccine mediated cell activation may trigger this cycling with no requirement for gene activation. Transcript analysis at a time point earlier than 24 h would help to elucidate the kinetics of the *cd86* gene. In contrast to *cd86*, the transcriptional response profiles for CD14, CD40, and CCR7 following treatment with the mRNA vaccine matched the protein expression profiles detected by flow cytometry in the PTE derived cells. Administration of the mRNA vaccine also led to significant induction of chemokines and cytokines locally at the injection site in mice and in MIMIC®-PTE modules. In the mice, the CXCR3-ligands CXCL9, CXCL10 and CXCL11 whose pleiotropic functions include stimulation of monocytes/macrophages, T cells, NK cells, and dendritic cells migration showed the most pronounced up-regulation among the chemokines, up-regulation that was reflected in the transcriptional analysis of human MIMIC® modules. In the human MIMIC® sentinel markers for immune cell activation were all up-regulated, including IL-12(p40), IL-12(p70), IFN-α, and TNF-α.

When comparing responses to the mRNA vaccine between MIMIC®-PTE modules and mice after intradermal injection there is a fundamental difference between the two that must be addressed. After innate stimulation immune cells and specifically dendritic cells in vivo capture and process antigen, mature and are activated, and migrate to the lymph node to prime the adaptive immune system. Consequently the gene signature of activation will gradually be lost in the injection site and appear in the draining lymph node. In the MIMIC® immune cells, which consist primarily of antigen presenting cells, respond to stimuli like mRNA vaccines to activate and mature in place because migration is not possible in this system. When evaluating the MIMIC®-PTE modules responding APCs are examined 24 h after treatment. Consequently MIMIC®-PTE modules may demonstrate innate response characteristics found in both the skin and in the dLN of the mouse model. Overall, however, while there is some correlation between the MIMIC® dataset and the mouse datasets, specifically the injection site 6 h post-injection and the dLN 24 h post injection, differences are evident and may be driven by the species tested, the in vitro versus in vivo models, or unidentified reasons.

## Conclusions

The translational approach used in this pre-clinical assessment into the basic mechanisms of self-adjuvantation from the mRNA vaccine allowed the identification of the mechanism of action by which the vaccine exerts its effect in humans and mice. In both species the vaccine acts through cellular RNA sensors, driving maturation and activation of immune cells as well as production of cytokines and chemokines known to attract and activate key players of the innate and adaptive immune system. In addition, because this approach could simultaneously be applied to both the in vitro human MIMIC® and in vivo mouse studies, correlative or divergent responses between the two species and two types of models were identified. Based on consistency between the two species in phenotypic, cytokine/chemokine, and transcriptional response to mRNA vaccine treatment, the mechanism of action of the adjuvant activity of this mRNA vaccine appears to be relatively conserved or at least convergent between the two species indicating that the innate immune stimulation from mRNA vaccines seen in mice translates to the human system. In addition, the results demonstrate that the MIMIC® system can be useful in preclinical evaluations of innate immune response to mRNA vaccines, with the potential identification of relevant pathways only evident in humans while demonstrating great similarity in the overall activation profile found in mouse studies.

## Additional files

**Additional file 1: Fig. S1.** Components and assembly of the PTE module for innate responses. (A) Top panel—in vivo monocytes continuously emigrate from the blood into peripheral tissues with a half-life in the blood of ~1 day. Bottom panel: In vitro generation of cytokine derived DCs from monocytes involves the addition of IL-4 and GM-CSF and culture for 7–11 days. (B) Schematic of the components of a MIMIC-PTE module. In the 3D PTE, differentiation occurs in hours to about 2 days triggered by migration into and out of (reverse migration) through the endothelium: a process reminiscent of the movement of cells from tissues into lymphatic vessels.

**Additional file 2: Fig. S2.** Treatment with R848, higher mRNA vaccine doses drives decreased APC cell viability and recovery. (A) Leukocyte subset (CD3+ or CD19+): Largely unaffected by mRNA vaccine treatment (small drop in elderly donors). (B) APC subset of cells (HLADR+): Fewer recovered cells from R848, dose-dependent drop from mRNA vaccine. Mean ± SEM are shown for n = 24 subjects examined in MIMIC® modules.

**Additional file 3: Fig. S3.** mRNA vaccine dose-driven maturation of APCs. (A) APC maturation was measured as a shift from CD14^high^/HLADR^high^ to CD14^low^/HLADR^high^ (seen in the flow panes in the top left) and up-regulation of HLA-DR (Fig. 1b). (B) TLR 7/8 activation drives APC maturation (R848). A dose-dependent maturation driven by the mRNA vaccine in APCs is evident over the range from 5 to 50 μg/10^6^ cells. Mean ± SEM are shown for n = 24 subjects examined in MIMIC® modules.

**Additional file 4: Fig. S4.** Dose-driven activation of APCs by mRNA vaccine. GMFI increases of (A) CCR7 (B) CD40, (C) CD25 and frequency of cells expressing the activation markers (D) CCR7, (E) CD40, and (F) CD25 are all greatly enhanced by both R848 and the mRNA vaccine. A dose-dependent maturation driven by the mRNA vaccine in APCs is evident over the range from 5 to 50 µg/10^6^ cells. Mean ± SEM are shown for n = 24 subjects examined in MIMIC® modules.

**Additional file 5: Fig. S5.** The mRNA vaccine (5–50 µg/10^6^ cells) and R848 (5 µg/ml) drive cytokine production indicative of TLR activation (A) IFNα, (B) IL-12(p70), (C) IL-6. Mean ± SEM are shown for n = 24 subjects examined in MIMIC® modules.

**Additional file 6: Fig. S6.** The ten most significant up-regulated immune pathways following stimulation by the mRNA vaccine. (A) Pathway map based off a gene-set enrichment pathway describes the most significantly associated pathways with the mRNA vaccine treatment. Analysis performed with Ingenuity pathway analysis software. (B) Visualizations of network associations.

## Abbreviations

mRNA: messenger ribonucleic acid
RLRs: RIG-I-like receptors
TLRs: Toll-like receptors
CLRs: C-type lectin receptors
mDCs: myeloid dendritic cells
pDCs: plasmacytoid dendritic cells
MIMIC®: Modular Immune In vitro Construct
PTE: Peripheral Tissue Equivalent
TW-PTE: Transwell Peripheral Tissue Equivalent
NHP: non-human primate
PRRs: pattern recognition receptors
ssRNA: single-stranded RNA
dsRNA: double-stranded RNA
HIV: human immunodeficiency virus
IL: interleukin
JNK: c-jun N-terminal kinase
DNA: deoxyribonucleic acid
HA: hemagglutinin
dLN: draining lymph node
ID: intradermal
PBMCs: peripheral blood mononuclear cells
APCs: antigen presenting cells
CD: cluster of differentiation
ANOVA: one-way analysis of variance
MHC: major histocompatibility complex class
Th: T helper
Tr1: type 1 regulatory
MAP kinase: mitogen-activated protein kinase
CXCR: CXC chemokine receptor
NLR: NOD-like receptors
