# Supplementary figures and images for: Adjuvant effects of a sequence-engineered mRNA vaccine: translational profiling demonstrates similar human and murine innate response
Source: J Transl Med. 2017 Jan 3;15:1. doi: 10.1186/s12967-016-1111-6 (PMC5210268; doi:10.1186/s12967-016-1111-6)

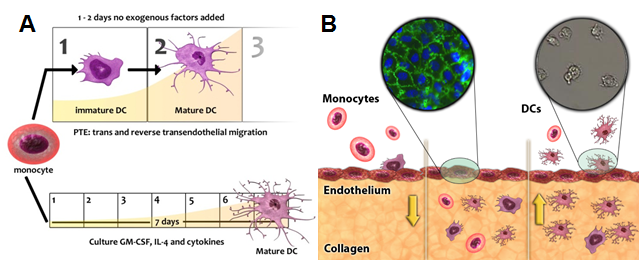

Supplement: Supplementary file 1 — Additional file 1: Fig. S1. Components and assembly of the PTE module for innate responses. (A) Top panel—in vivo monocytes continuously emigrate from the blood into peripheral tissues with a half-life in the blood of ~1 day. Bottom panel: In vitro generation of cytokine derived DCs from monocytes involves the addition of IL-4 and GM-CSF and culture for 7–11 days. (B) Schematic of the components of a MIMIC-PTE module. In the 3D PTE, differentiation occurs in hours to about 2 days triggered by migration into and out of (reverse migration) through the endothelium: a process reminiscent of the movement of cells from tissues into lymphatic vessels. [file 12967_2016_1111_MOESM1_ESM.tif]

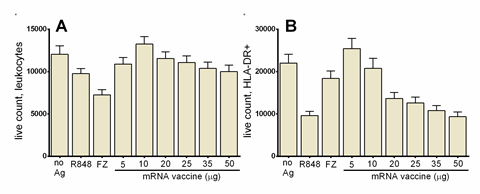

Supplement: Supplementary file 2 — Additional file 2: Fig. S2. Treatment with R848, higher mRNA vaccine doses drives decreased APC cell viability and recovery. (A) Leukocyte subset (CD3+ or CD19+): Largely unaffected by mRNA vaccine treatment (small drop in elderly donors). (B) APC subset of cells (HLADR+): Fewer recovered cells from R848, dose-dependent drop from mRNA vaccine. Mean ± SEM are shown for n = 24 subjects examined in MIMIC® modules. [file 12967_2016_1111_MOESM2_ESM.tif]

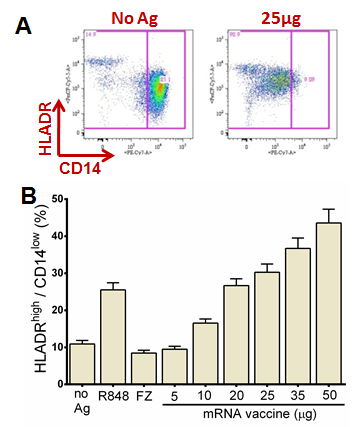

Supplement: Supplementary file 3 — Additional file 3: Fig. S3. mRNA vaccine dose-driven maturation of APCs. (A) APC maturation was measured as a shift from CD14high/HLADRhigh to CD14low/HLADRhigh (seen in the flow panes in the top left) and up-regulation of HLA-DR (Fig. 1b). (B) TLR 7/8 activation drives APC maturation (R848). A dose-dependent maturation driven by the mRNA vaccine in APCs is evident over the range from 5 to 50 μg/106 cells. Mean ± SEM are shown for n = 24 subjects examined in MIMIC® modules. [file 12967_2016_1111_MOESM3_ESM.png]

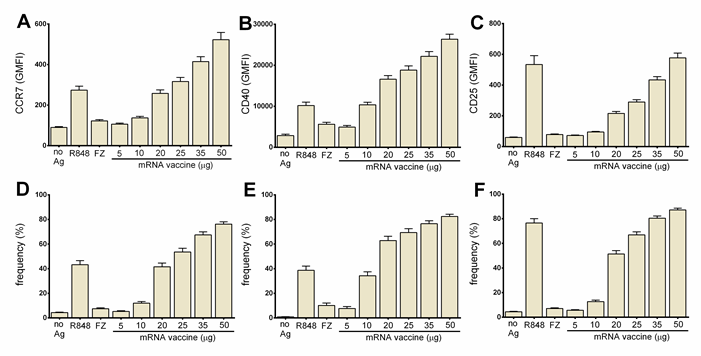

Supplement: Supplementary file 4 — Additional file 4: Fig. S4. Dose-driven activation of APCs by mRNA vaccine. GMFI increases of (A) CCR7 (B) CD40, (C) CD25 and frequency of cells expressing the activation markers (D) CCR7, (E) CD40, and (F) CD25 are all greatly enhanced by both R848 and the mRNA vaccine. A dose-dependent maturation driven by the mRNA vaccine in APCs is evident over the range from 5 to 50 µg/106 cells. Mean ± SEM are shown for n = 24 subjects examined in MIMIC® modules. [file 12967_2016_1111_MOESM4_ESM.tif]

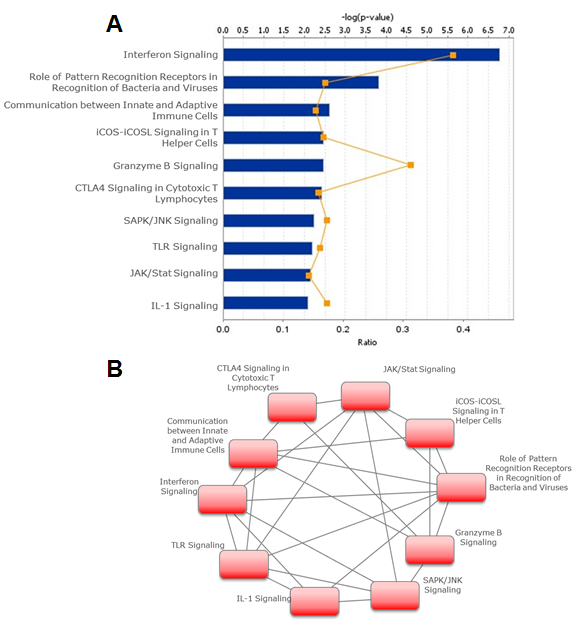

Supplement: Supplementary file 6 — Additional file 6: Fig. S6. The ten most significant up-regulated immune pathways following stimulation by the mRNA vaccine. (A) Pathway map based off a gene-set enrichment pathway describes the most significantly associated pathways with the mRNA vaccine treatment. Analysis performed with Ingenuity pathway analysis software. (B) Visualizations of network associations. [file 12967_2016_1111_MOESM6_ESM.tif]
